# Genome-wide CRISPR/Cas9 screening in human iPS derived cardiomyocytes uncovers novel mediators of doxorubicin cardiotoxicity

**DOI:** 10.1038/s41598-021-92988-1

**Published:** 2021-07-06

**Authors:** Valerie Sapp, Aitor Aguirre, Gayatri Mainkar, Jeffrey Ding, Eric Adler, Ronglih Liao, Sonia Sharma, Mohit Jain

**Affiliations:** 1 Department of Medicine, University of California, San Diego, San Diego, CA USA; 2 Department of Pharmacology, University of California, San Diego, San Diego, CA USA; 3 Department of Medicine, Stanford University, Palo Alto, USA; 4 La Jolla Institute for Immunology, San Diego, CA USA; 5 Present Address: Division of Developmental and Stem Cell Biology, Institute for Quantitative Health Science and Engineering, Michigan State University, East Lansing, MI USA; 6 Present Address: Department of Biomedical Engineering, Michigan State University, East Lansing, MI USA

**Keywords:** Drug safety, Toxicology, High-throughput screening, Functional genomics, Pluripotent stem cells, Stem-cell differentiation, CRISPR-Cas9 genome editing, Cardiomyopathies

## Abstract

Human induced pluripotent stem (iPS) cell technologies coupled with genetic engineering now facilitate the study of the molecular underpinnings of disease in relevant human cell types. Application of CRISPR/Cas9-based approaches for genome-scale functional screening in iPS-derived cells, however, has been limited by technical constraints, including inefficient transduction in pooled format, loss of library representation, and poor cellular differentiation. Herein, we present optimized approaches for whole-genome CRISPR/Cas9 based screening in human iPS derived cardiomyocytes with near genome-wide representation at both the iPS and differentiated cell stages. As proof-of-concept, we perform a screen to investigate mechanisms underlying doxorubicin mediated cell death in iPS derived cardiomyocytes. We identified two poorly characterized, human-specific transporters (*SLCO1A2*, *SLCO1B3*) whose loss of function protects against doxorubicin-cardiotoxicity, but does not affect cell death in cancer cells. This study provides a technical framework for genome-wide functional screening in iPS derived cells and identifies new targets to mitigate doxorubicin-cardiotoxicity in humans.

## Introduction

The discovery of human cellular plasticity and the reprogramming of adult somatic cells to induced pluripotent stem (iPS) cells have ushered in new tools and approaches for interrogating human biology^1,2^. iPS cells may be readily differentiated into a number of human cell types^3–5^, including terminally differentiated cells not typically amenable to isolation and culture, such as specialized neurons or cardiomyocytes^5–8^. iPS derived cells have provided invaluable insights into biological processes and mechanisms underlying patient specific characteristics^9,10^, cell type specific differentiation^3–5^, human disease states^11–13^, and drug toxicity^14–16^.

Facile genetic manipulation particularly at genome-wide scale is crucial to leveraging the discovery potential of iPS cells. Traditionally, however, human iPS cells have proven resistant to conventional targeting methods relative to transformed human cells or even mouse iPS cells^17–20^. Recently, application of clustered regularly interspersed palindromic repeat (CRISPR)/Cas9 approaches has greatly improved the efficiency with which single functional mutations may be introduced in a wide range of organisms and cell types, including human iPS lines^21–26^. Coupled with large scale, pooled libraries of CRISPR/Cas9 reagents for gene disruption iPS cells now, theoretically, allow for the genome-scale functional study in relevant primary human cell types^27,28^. To date, efforts to apply genome-wide CRISPR/Cas9 to forward genetic screens in iPS derived cells have been limited. A key challenge for these approaches has been the efficient infection of iPS cells with virus encoding CRISPR/Cas9 reagents^29^. At too high an infection rate, iPS cells are likely to be infected with multiple CRISPR/Cas9 reagents, resulting in simultaneous disruption of multiple genes, thereby complicating deconvolution of results^29^. A lower than optimal infection rate, however, precludes interrogation of the whole genome^29^. In addition, it remains to be determined whether infection with pooled lentiviral reagents required for genome-wide CRISPR sgRNA expression would alter either iPS cell stemness or differentiation capacity. Finally, it is unclear whether these approaches may be applied to reveal novel human-specific mechanisms underlying cardiomyocyte phenotypes.

Herein, we find that reverse transduction greatly enhances lentiviral infection of human iPS cells. Using this method, we optimize approaches for CRISPR/Cas9 forward genetic screening in human iPS derived cells and achieve near genome-wide representation of CRISPR sgRNAs in both stem cells and differentiated cardiomyocytes, without altering makers of stemness or differentiation, respectively. Finally, in a proof of concept genome-wide CRISPR/Cas9 screen, we uncover cell-autonomous, human-specific mediators of doxorubicin induced cardiotoxicity in iPS derived cardiomyocytes, further highlighting the discovery potential of these approaches. This study presents an accessible strategy for CRISPR/Cas9 mediated screening in human iPS derived cells at genome scale. Application of these techniques will facilitate discovery of novel to human biology and disease mechanisms in relevant differentiated cell types.

## Results

### Lentiviral targeting of human iPS cells

**Figure 1:**
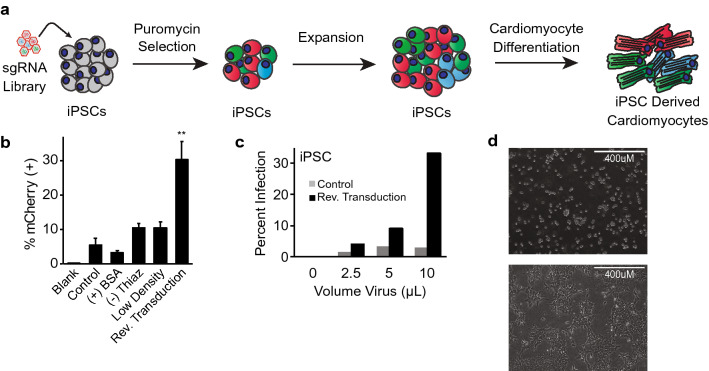
Optimization of CRISPR/Cas9 transfer in human iPS cells. (**a**) Schematic for forward genetic CRISPR/Cas9 screening in induced pluripotent stem (iPS) derived cardiomyocytes. (**b**) Optimization of infection efficiency with mCherry expressing virus. Cells were treated with no virus (blank), standard infection protocols (control), addition of 1% BSA during standard infection (+ BSA), removal of the ROCK inhibitor thiazovivin during plating (− Thiaz), plating at half density prior to standard infection (low density) or use of reverse transduction (n = 2 per condition, student’s T-test, **: *p* < 0.01). (**c**) Infection efficiency in iPS cells using standard (control) or reverse transduction approaches (n = 2 per condition). (**d**) Bright-field image of iPS cells during plating (top) and 24 h after plating (bottom).

Application of genome-wide CRISPR/Cas9 approaches in pooled human iPS cells necessitates efficient transduction at the iPS stage, followed by selection, expansion, and differentiation into relevant human cell types for study (Fig. 1a). Chief among these steps is optimized infection of iPS cells with lentivirus encoding CRISPR/Cas9 reagents at an infection rate of ~ 30%, to allow for genome-wide representation while statistically limiting the likelihood of incorporation of multiple sgRNAs into a single cell^29^. Using a lentiviral mCherry reporter system, we found that human iPS cells were resistant to viral infection using standardized infection protocols previously utilized in immortalized and/or transformed non-iPS cell lines^30–32^, with less than 10% infection rate (Fig. 1b). Application of methods that have been reported to enhance viral infection, including exposure to increased concentration of bovine serum albumin (BSA)^33^, or low-density culture conditions^32,34^, did not significantly improve lentiviral infection in human iPS cells, nor did removal of ROCK inhibitor thiazovivin (Fig. 1b). Prior work has suggested that viral infection of cells during plating, also known as ‘reverse transduction’ may enhance infection rates^35,36^. Indeed, we found that reverse transduction greatly increased infection efficiency relative to control methods (Fig. 1b, Supplementary Fig. 1).

We next determined whether the enhanced viral infectivity of iPS cells observed with reverse transduction translated to viral infection with a CRISPR/Cas9 lentiviral system. Using a well described lentiviral system expressing Cas9 and sgRNA in single vector^28^, human iPS cells were infected with increasing virus volume. Reverse transduction resulted in a marked increase in viral infection of greater than sixfold relative to standard infection protocols (Fig. 1c). Enhanced lentiviral infection was also noted in human embryonic stem (H1) cells (Supplementary Fig. 1). Notably, reverse transduction results in greater cellular surface area exposure during viral infection versus traditional adherent cell culture, particularly as iPS cells tend to grow in colonies with cell–cell contact occupying a significant portion of surface area (Fig. 1d), potentially accounting for the improved infection efficiency with reverse transduction.

### Infection of human iPS cells with genome-wide CRISPR/Cas9 sgRNA library

**Figure2:**
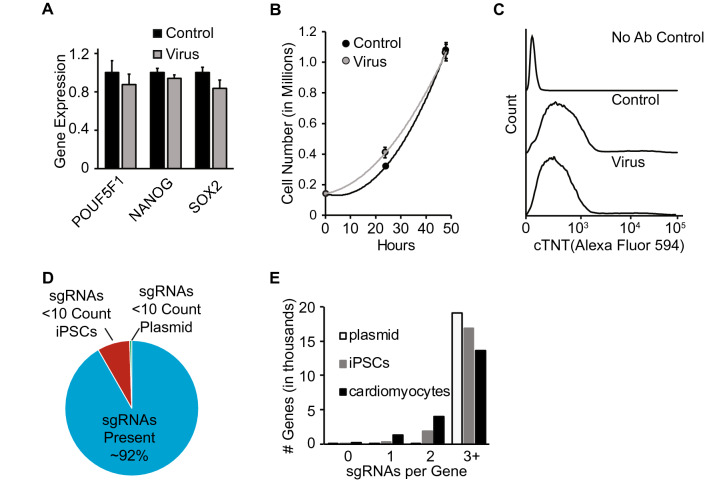
Phenotypic resilience in pooled CRISPR/Cas9 library infected iPS cells. Uninfected iPS cells (control) or those transduced with CRISPR/Cas9 library (virus) were assessed for (**a**) gene expression of pluripotency markers (n = 3 per condition), (**b**) proliferative potential (n = 3 per condition), and (**c**) cardiac troponin T (cTNT) protein expression after differentiation to cardiomyocytes. (**d**) Percent of CRISPR/Cas9 library sgRNAs present in iPS cells at 10 read-counts or higher. (**e**) Number of sgRNAs per gene present in plasmid, iPS cells, and iPS derived cardiomyocytes.

We next determined whether viral infection or expression of a CRISPR/Cas9 library in human iPS cells alters cellular state. Infection of human iPS cells with lentivirus expressing Cas9-sgRNA followed by puromycin selection for guide expression did not alter expression of key transcriptional markers of pluripotency, POU5F1, Nanog or Sox2 (Fig. 2a, Supplementary Fig. 2) or iPS cell proliferation rate (Fig. 2b, Supplementary Fig. 2) relative to uninfected cells. Following differentiation into cardiomyocytes, CRISPR/Cas9 infected iPS cells exhibited robust expression of the cardiac-specific markers cardiac troponin T (cTNT) (Fig. 2c) and myosin light-chain 2 (MLC2) (Supplementary Fig. 2), similar to uninfected cells and clear rhythmic contraction (Supplementary Video 1) consistent with normal cardiomyocyte physiology. These data suggest that gross markers of cellular physiology are unchanged at the iPS cell or differentiated cell stage with introduction of whole-genome CRISPR/Cas9 reagents. Additionally, we performed Karyotype analysis on uninfected iPS cells (Supplementary Fig. 3) and confirmed that there were no clonal or structural abnormalities that would introduce systemic bias into a whole-genome CRISPR/Cas9 screen.

We next sought to determine whether human iPS cells infected with a genome-wide CRISPR/Cas9 library retain full coverage sgRNA expression. Human iPS cells (9 × 10^7^ cells) were infected with lentivirus encoding a well-described genome-wide CRISPR/Cas9 library containing ~ 75,000 total sgRNAs targeting ~ 19,000 human genes^28^. Following puromycin selection and 10 × expansion, human iPS cells retained robust expression of the sgRNA library, with loss of only 8% of sgRNAs relative to the original plasmid library (Fig. 2d). Lost sgRNAs were found to target genes essential to cellular survival and proliferation, including ribosomal, mRNA processing and cell cycle genes (Supplementary Fig. 4). iPS cells were subsequently differentiated into cardiomyocytes and the sgRNA library again sequenced. At the cardiomyocyte stage, sgRNAs targeting only a small fraction of genes were lost relative to the iPS stage, with continued expression of at least 3 sgRNAs targeting greater than 13,000 genes (Fig. 2e, Supplementary Fig. 4), enabling near genome-wide representation required for comprehensive forward genetic screening.

### Genome-wide CRISPR/Cas9 screening for modulators of cardiotoxicity

**Figure 3:**
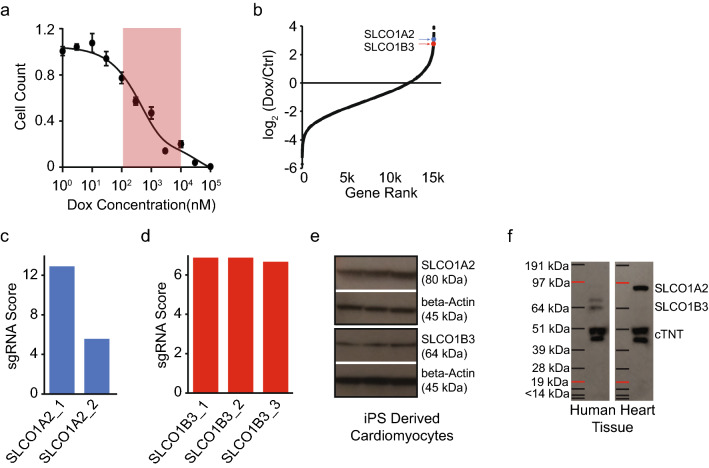
Genome-scale CRISPR/Cas9 screen in iPS derived cardiomyocytes for mediators of doxorubicin-toxicity. (**a**) Dose response curve for doxorubicin-toxicity in iPS derived cardiomyocytes (circulating concentration range highlighted in red, n = 8 per condition). (**b**) Distribution of fold change scores in doxorubicin (dox) versus control (ctrl) treated cardiomyocytes. SLCO1A2 and SLCO1B3 indicated by blue and red arrows, respectively. (**c**), and (**d**) Enrichment scores for individual sgRNAs targeting SLCO1A2 and SLCO1B3. (**e**) Western-blot for protein expression of SLCO1A2 and SLCO1B3 in human iPS derived cardiomyocytes. Full-length blots are included in Supplementary Figure 10a–b. (**f**) Western-blot for protein expression of SLCO1A2 and SLCO1B3 in human heart tissue. Full-length blots are included in Supplementary Figure 10c–d.

Cardiotoxicity remains among the most significant impediments for new drug approval, particularly among many anti-cancer agents^37^. While a number of potential contributors may lead to drug associated cardiotoxicity, the fundamental mechanisms underlying chemotherapy related cardiotoxicity in humans still remain unclear^37,38^. Recently, iPS derived cardiomyocytes have been demonstrated to be a particularly useful system for probing mechanisms of cardiotoxicity associated with doxorubicin (DOX), a common first line chemotherapy used in breast cancer^14,39^. As proof of concept, we applied our whole-genome CRISPR/Cas9 knockout screening approach to identify new cell-autonomous mechanisms of DOX induced cardiotoxicity. Our rationale was that cardiomyocytes containing knockout genes that facilitate DOX-toxicity would have higher resistance to the chemotherapeutic, and thus would enrich over time due to prolonged survival. Exposure of iPS derived cardiomyocytes to DOX resulted in significant cell death at reported circulating concentrations (Fig. 3a)^14,40,41^. We next evaluated the impact of CRISPR/Cas9 library infection on DOX-toxicity in iPS derived cardiomyocytes (Supplementary Fig. 2) and found a small, but significant increase in survival in library infected cells. This increase in survival likely represents to the protective effect of sgRNAs targeting genes involved in DOX-toxicity, we thus selected representative concentration of 3 μM for genetic screening.

iPS cells were transduced with a whole-genome CRISPR/Cas9 library, selected, expanded, and differentiated into highly pure cardiomyocytes and exposed to DOX for 72 h. gDNA was then collected for PCR amplification of sgRNA guides and sequencing. Gene ontology analysis revealed DOX-toxicity affected processes related to iron metabolism and mitochondrial function (Supplementary Fig. 5), both key biological processes previously implicated in DOX-toxicity^42^. Of note, we found significant enrichment for membrane protein transporters (Supplementary Fig. 5), particularly among SLC solute carrier family, whose silencing protected against DOX-cardiotoxicity (Fig. 3b). Examination of individual sgRNAs found multiple sequence-independent guides targeting *SLCO1A2* (solute carrier organic anion transporter family member 1A2, also known as OATP1; *p* = 0.017) and *SLCO1B3* (solute carrier organic anion transporter family member 1B3, also known as OATP8 and LST2; *p* = 0.0012), two related organic anion transporters (Fig. 3c, d)^43^. Both SLCO1A2 and SLCO1B3 protein were found to be expressed in in human iPS derived cardiomyocytes (Fig. 3e) and adult human hearts (Fig. 3f). We therefore hypothesized that these transporters may be essential for DOX-cardiotoxicity.

### SLCO1A2 is essential for doxorubicin cardiotoxicity

**Figure 4:**
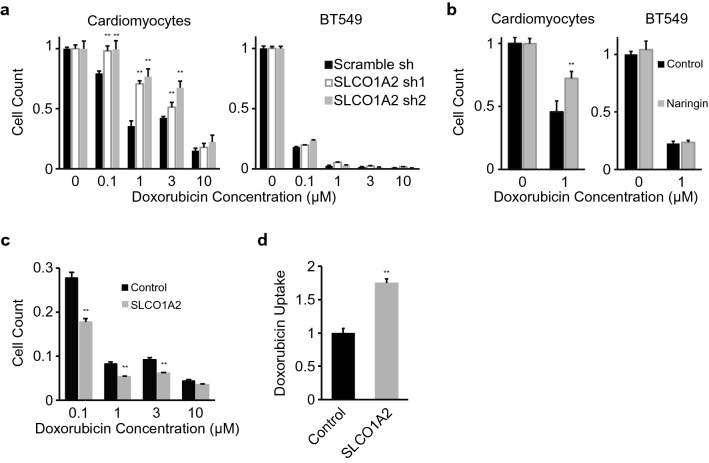
SLCO1A2 mediates doxorubicin-toxicity in iPS derived cardiomyocytes. (**a**) Dose response for doxorubicin toxicity in cells expressing control Scramble shRNA, or two sequence independent shRNAs targeting SLCO1A2 in iPS derived cardiomyocytes or BT549 breast cancer cells (**: *p* < 0.01 vs scramble shRNA). (**b**) Doxorubicin-toxicity in cells treated with the SLCO1A2 inhibitor naringin in iPS derived cardiomyocytes or BT549 cancer cells (**: *p* < 0.01). (**c**) Doxorubicin toxicity (n = 8 per condition) and (**d**) uptake (n = 3 per condition) in cells over-expressing SLCO1A2 (**: *p* < 0.01). (**a**–**d**) *p* from student’s T-test.

After leveraging CRISPR/Cas9 based screening to uncover SLCO1 family transporters as novel disease mediators in iPS derived cardiomyocytes, as a proof of concept we further determined the role of SLCO1A2 in doxorubicin induced cardiotoxicity. We selected two sequence-independent shRNAs to reduce the expression of SLCO1A2 in iPS derived cardiomyocytes (Supplementary Fig. 6), utilizing an approach orthogonal to the CRISPR/Cas9 based method where SLCO1A2 initially highlighted as a mediator of DOX-cardiotoxicity. Loss of SLCO1A2 protected against cell death within the reported circulating range of DOX (0.1–3 μM) but not at higher concentrations (Fig. 4a), suggesting that other low-affinity transporters may also be capable of DOX uptake at elevated concentration. We further confirmed that SLCO1A2 knockdown also impacted DOX mediated cell death in hES derived cardiomyocytes at the lower end of the reported circulating range (0.1–0.3 μM) (Supplementary Fig. 7). We next evaluated whether target cancer cells similarly utilize SLCO1A2 for transport of DOX. Silencing of SLCO1A2 did not affect DOX mediated cell death in BT549 breast cancer cells at reported circulating (Fig. 4a) or even at lower (Supplementary Fig. 8) concentrations, suggesting that other key transporters may mediate DOX-related cell death in BT549 breast cancer cells^44^. Pharmacologic inhibition of SLCO1A2 with the small molecule naringin phenocopied genetic silencing, and similarly protected against DOX-cardiotoxicity in cardiomyocytes, but not breast cancer cells (Fig. 4b)^45^. To further validate the role of specific human transporters in DOX toxicity, SLCO1A2 was overexpressed (Supplementary Fig. 9). Overexpression of SLCO1A2 enhanced DOX mediated cell death (Fig. 4c) and directly mediated uptake of DOX (Fig. 4d), suggesting a direct role for SLCO1A2 mediated transport in DOX-toxicity.

## Discussion

In the present study we establish and apply approaches for genome-scale CRISPR/Cas9 genetic screening in iPS derived cells. We identify and optimize conditions for efficient lentiviral infection of cells at the iPS state, which allows for infection with genome-wide sgRNA libraries. We further demonstrate that CRISPR/Cas9 library infected iPS cells maintain their pluripotent state and retain their characteristics of proliferation and differentiation. Library infected cells retained cell-type specific physiology upon differentiation, including cell-type specific protein expression and function, such as contraction/relaxation in differentiated cardiomyocytes, despite expression of sgRNAs targeting > 91% of the genome. Collectively, these advances enable pooled, genome-scale, forward, loss of function screening in human cells. As a proof of concept, we apply these described approaches to uncover previously unrecognized modulators of drug related cardiotoxicity and identify SLCO1A2 as a key transport mechanism underlying DOX toxicity specifically in human iPS derived cardiomyocytes.

The differentiation of iPS cells affords a unique opportunity to study development and disease in the relevant human cell types and has proven especially useful for difficult to isolate cell types such as cardiomyocytes or neurons^17,46,47^. Until recently, insights into cardiomyocyte- or neuron-specific biology were largely limited to rodent and other small animal models^10^. While certainly valuable as model systems, lower vertebrates including mice and rats have well described and key genetic, molecular and physiological discrepancies relative to humans potentially limiting discovery and slowing the translation of findings from rodents to man^48–51^. This limitation is underscored in our study: the transporter proteins SLCO1A2 and SLCO1B3, identified here as mediators of DOX-cardiotoxicity, do not have direct one-to-one orthologs in commonly used small model organisms^43^. As such, the importance of these transporters in DOX-cardiotoxicity would not have been uncovered using mouse or rat models, highlighting the importance of functional genomics approaches specifically in human cells. Moreover, given that these transporters are found to mediate cellular toxicity uniquely in cardiomyocytes rather than in typically studied transformed cell lines, the importance of iPS derived cells is further noted. Importantly, the approaches and advantages of unbiased CRISPR/Cas9 based discovery in iPS derived cells outlined here are readily transferrable to other iPS based systems and cell types of interest.

To date, CRISPR/Cas9 approaches have been largely applied to iPS derived cells to examine a subset of human genes, such as long non-coding RNAs or largely focused upon iPS cell biology and cell-type specific differentiation^52–54^, rather than unbiased analysis of the whole genome in differentiated cell types, which necessitates optimized infection rates. Other studies, have similarly centered on CRISPR/Cas9 screening in selected iPS cells or specialized iPS derived cells, such as neural progenitor cells, that are proliferative and typically more amenable to viral infection^55–57^. Our optimized methods in human iPS cells indicate that ‘reverse-transduction’ approaches greater improve lentiviral infection efficiency for a pooled CRISPR/Cas9 library. iPS cells typically grow in densely packed colonies with minimal surface area exposed thereby limiting viral contact. Reverse transduction allows for interaction of viral particles with dissociated cells in suspension, during which time the exposed surface area is significantly increased. This reverse transduction approach closely resembles the suspension infection techniques first used with lentiviral infection of human embryonic stem cells^58,59^, and similar techniques have been previously employed to increase adenoviral infection efficiency in human cells^35,36^.

In applying the described CRISPR/Cas9 approaches in iPS derived cells, we have also uncovered and validated a role for the cell surface transporters SLCO1A2 and SLCO1B3 in doxorubicin induced cardiotoxicity. Whereas the transporters required for efflux of doxorubicin and related chemotherapies have been described^60^, to date, the transport systems that promote uptake of doxorubicin under physiologic concentrations into human cardiomyocytes have remained unclear. While SLCO1A2 and SLCO1B3 likely mediate doxorubicin uptake and clearance^61^ at reported circulating concentrations^14,40,41^, it is notable that cardiomyocytes and cancer cells exhibit distinct uptake kinetics^62^ and we find that other transport systems likely mediate doxorubicin uptake into breast cancer cells. Given this selectivity for doxorubicin uptake via SLCO1A2 in cardiomyocytes, this transporter represents an intriguing drug target to mitigate doxorubicin induced cardiotoxicity, while preserving the drug’s chemotherapeutic effect. Taken together our results demonstrate the utility of an optimized functional genomics approach in iPS derived cells for novel human discovery.

## Methods

Detailed methods are included in the Supplementary Information appendix, Materials and Methods.

### Cell culture and reagents

BT549 breast cancer cells were obtained from ATCC. HEK293T cells were obtained from Sonia Sharma(LJI), H1ESC line was obtained from Aitor Aguirre(UCSD), and IPSL1 was a obtained from Eric Adler(UCSD). Cells were maintained as described in the Supplementary Methods and cardiomyocyte differentiation was performed was described previously^8^. Antibodies were obtained according to the following: Anti-cTnT primary antibody (ThermoFisher MA5-12960), Anti-SLCO1A2 (abcam ab221804), Anti-SLCO1B3 (abcam ab224064), Anti-βActin primary antibody (Cell Signaling 3700S), Ant- Myosin Light Chain (abcam ab89594), HRP Conjugated Anti Rabbit (Enzo ADI-SAB-300J), HRP Conjugated Anti Mouse (Cell Signaling 7076P2), and Alexa Fluor 594 anti-mouse secondary antibody (ThermoFisher A-21203). Doxorubicin (Sigma D1515) and Narignin (71162) were obtained from Sigma Aldrich.

### Heart tissue

Heart tissue protein lysate was obtained from explanted human cardiac tissue. All research was conducted in accordance with relevant guidelines and regulations. All human subjects work was conducted under approval of the UCSD Institutional Review Board (IRB) and Human Research Protection Program and informed consent was obtained from all subjects.

### CRISPR/Cas9 screening

The Brunello LentiCRISPR plasmid library^28^ was obtained from Addgene (Cat # 73178), amplified according to established Broad Institute GPP Protocol, and virus was subsequently produced using HEK293T cells as described in the Supplementary Methods. The multiplicity of infection for each cell type was determined empirically as previously described^27^. For the genome wide screening, 87.5 million iPS cells were infected with the Brunello library at an efficiency rate of 30% and selected for lentiviral incorporation using puromycin. CRISPR/Cas9 library iPS cells were then differentiated to cardiomyocytes as described previously^8^. For the doxorubicin (DOX) treatment, iPS cells were treated with 3 μM doxorubicin for 72 h. DNA was collected for sgRNA sequencing from iPS cell, differentiated cardiomyocyte and doxorubicin treated conditions. Data was processed using Mageck analysis software and the RIGER algorithm to analyze sgRNA and gene level enrichment in doxorubicin treated versus no-doxorubicin control^63,64^.

### SLCO1A2 validation

Plasmid encoding scramble or SLCO1A2 shRNA were purchased from VectorBuilder and lentivirus was generated as in the Supplementary Methods. iPS cells, ES cells and BT549 breast cancer cells were infected in media containing polybrene during plating. Cells expressing the shRNA cassette were selected using puromycin. For transient over-expression, HEK cells were transfected with plasmid encoding for SLCO1A2 or were mock transfected without plasmid for control. For stable over-expression HEK cells were infected with virus encoding SLCO1A2 in media containing polybrene during plating. iPS derived cardiomyocytes and Bt549 breast cancer cells were treated with SLCO1A2 inhibitor Naringin at 50 μM or vehicle control. Doxorubicin toxicity was assessed using nuclei counting, as described in Supplementary Methods. Doxorubicin uptake was detected using mass-spectrometry based measures of cell lysate, as outlined in the Supplementary Methods.

### Statistical analysis

Statistical significance was determined using students T-test or linear regression using R statistical analysis software. Data is illustrated as mean +/− SEM. GraphPad PRISM software was used to fit a dose–response curve to doxorubicin toxicity data using the bell-shape model.

## Supplementary information

Supplementary Information 1.

Supplementary Information 2.

Supplementary Video 1.

Supplementary Information 3.
