# Supplementary material for: Genome-wide CRISPR/Cas9 screening in human iPS derived cardiomyocytes uncovers novel mediators of doxorubicin cardiotoxicity
Source: Sci Rep. 2021 Jul 6;11:13866. doi: 10.1038/s41598-021-92988-1 (PMC8260754; doi:10.1038/s41598-021-92988-1)
Supplement: Supplementary file 4 — Supplementary Information 3. [file 41598_2021_92988_MOESM4_ESM.pdf]

Genome-wide CRISPR/Cas9 Screening in Human iPS Derived Cardiomyocytes Uncovers Novel Mediators of Doxorubicin Cardiotoxicity

Valerie Sapp[1,2], Aitor Aguirre[1,5], Gayatri Mainkar[1,2], Jeffrey Ding[1,2],
Eric Adler[1], Ronglih Liao[3], Sonia Sharma[4], Mohit Jain[1,2,*]

1: Department of Medicine, University of California, San Diego; San Diego, CA

2: Department of Pharmacology, University of California, San Diego; San Diego, CA

3: Department of Medicine, Stanford University, Palo Alto,

4:La Jolla Institute for Immunology; San Diego, CA

5: Present address: Division of Developmental and Stem Cell Biology, Institute for Quantitative Health Science and Engineering, and Department of Biomedical Engineering, Michigan State University, East Lansing, MI

*: senior author

For Correspondence:
Mohit Jain, MD PhD
UC San Diego
mjain@ucsd.edu

Supplementary Materials and Methods:

Cell Culture:

Cells were maintained at 5% CO_2 at 37^oC in Heracell Vios incubator (Thermo Fisher) and all cells were maintained with penicillin/ streptomycin (Thermo Fisher 15140-122) except during plasmid transfection. IPSL1 was obtained from Eric Adler. All iPS and ESC lines were maintained in E8 Flex medium (Life Technologies A2858501) on Matrigel growth factor reduced matrix (Corning 354230) coated plates and passaged regularly using Relesr (StemCell Technologies 05872) and treated with 2 μM thiazovivin (Selleck S1459) during passaging. HEK293T cells were obtained from Sonia Sharma. HEK293-T cells were maintained in DMEM (Life Technologies 11995) supplemented with 10% FBS (Life Technologies 10437-028). BT549 breast cancer cells were obtained from ATCC and maintained in RPMI (Life Technologies 11875119) supplemented with 10% FBS and human recombinant insulin (Life Technologies 12585014). Cell counting was performed using a Moxi Z (Orflo MXZ001) automated cell counter. G-band based karyotyping analysis was performed by WiCell Institute (Madison, Wisconsin) on iPSL1 cells at passage 20 at a band resolution of 350-375 bands per haploid genome.

Cardiomyocyte Differentiation:

Differentiation was performed using the small molecule modulator strategy as previously described.[1] Briefly, differentiating cells were maintained in RPMI (Life Technologies 11875119) with B27 minus insulin (Life Technologies A1895601) from day 0-7 of differentiation and maintained in RPMI with B27 supplement (Life Technologies 17504044) from day7-15 of differentiation. Cells were treated with 10 μM GSK inhibitor, CHIR99021 (Selleck S1263) for 24 hours at day 0 of differentiation and with 2 μM WNT inhibitor, wnt-c59 (Selleck S7037), for 48 hours from day 3-5 of differentiation. Lactate selection was performed from day 15-25 of cardiomyocyte differentiation to purify cardiomyocytes as described previously.[2] Briefly, cardiomyocytes were grown in glucose free RPMI (Life Technologies 11879020) supplemented with B27 supplement and 5.5 mM lactate (Millipore Sigma L4263).

Brunello LentiCRISPR Library Amplification:

Brunello LentiCRISPR plasmid library was obtained from Addgene (Cat # 73178) and amplified according to established Broad Institute GPP Protocol (https://portals.broadinstitute.org/gpp/public/dir/download?dirpath=protocols/production&filename=pDNA_library_amplification_Jan2018.pdf).[3] Briefly, pooled Brunello LentiCRISPR was introduced into Endura electrocompetent cells (Lucigen 60242-2) using electroporation. After recovery, cells were plated on 500 cm^2 bioassay plates (Corning 06-443-22) with LB agar containing ampicillin and grown overnight at 30^oC. Cells were pelleted, and DNA was collected using ZymoPURE Maxiprep Kit (Zymo Research D4202).

Virus Production:

Virus was produced using HEK293T cells transduced with lentiviral expression plasmid and pMDa2 and psPAX2 lentiviral packaging plasmids. Briefly, cells were grown in Optimem (Thermo Fisher 31985062) media supplemented with Lipofectamine

(Thermo Fisher 11668030) and Plus Reagent (Thermo Fisher 11514015). Supernatant was collected 60 hours after transduction, filter sterilized using a Steriflip filtration device (Millipore Sigma SE1M003M00) according to manufacturer's instructions.

mCherry Virus Infection Rate Optimization:

Lentiviral construct expressing mCherry under the control of the EF1A promoter was obtained from Vector-Builder (VB160109-10005). iPS cells were infected with virus in media containing 8 µg/mL polybrene (AmericanBio AB01643-00001). Control, (+) BSA, (-) Thiaz and low-density conditions infection was performed 24 hours after plating, after cells had attached. For the reverse transduction condition, infection was performed during plating while cells were in suspension. Cells were allowed to proliferate for 72 hours after plating at which point cells were fixed with 4% PFA and stained with DAPI as described below. Fluorescent images were obtained on a Keyence BZX-700 Fluorescent Microscope at the UCSD Microscopy Core and cell counting was performed on stitched images for both mCherry and DAPI stained cells was performed using ImageJ particle analysis.

Brunello Library Infection rate optimization:

The multiplicity of infection for each cell type was determined empirically as described here.[4] Cells were plated at a density of 200,000 cells/well and treated with a range of volumes of concentrated Brunello LentiCRISPR virus, in media containing 8 µg/mL polybrene, either during plating or 24 hours after, once cells had attached. 48 hours after plating, cells were dissociated and re-plated into 6 well plate in quadruplicate. 24 hours after re-plating, cells in two replicate wells were treated with 0.4 µg/mL puromycin (Gibco A1113803), while two wells remained untreated as no-puromycin control. Cells were dissociated and counted when no cells remained uninfected control wells under puromycin selection.

Mock CRISPR Screening:

For assessment of pluripotency markers, proliferation potential and cardiomyocyte differentiation a mock screen at low (7 fold) representation was performed. Cells were dissociated to a single cell suspension with Accutase (StemCell Techologies 07922) and 1.2 million cells were plated into 3 wells of a 6-well plate. Each well was treated with 25 µL of Brunello lentiCRISPR virus for a pre-calculated infection efficiency of 30% in 8 µg/mL polybrene during plating. After 48 hours cells were re-plated for phenotypic assays. Cells were replated for cardiomyocyte differentiation at 500,000 cells per well of a 6 well plate and differentiation was performed as described above. Differentiated cardiomyocytes were assessed for cardiac markers or doxorubicin toxicity at day 30 post differentiation. Cells plated for pluripotency marker expression and proliferation assay were replated at 75,000 cells per well of a 6 well plate and assessed for pluripotency gene expression via qPCR after 72 hours.

CRISPR Screening:

iPS cells were dissociated to a single cell suspension using Accutase and plated at 12.5 million cells per flask into 8 T225 flasks (Thermo Fisher 159934) with 8µg/mL polybrene. Brunello lenti-CRISPR virus was as added to a pre-calculated infection rate

of 30% (600 µL virus per flask) into 7 flasks, leaving one flask uninfected as a control for puromycin selection. After 48 hours Brunello lentiCRISPR infected iPS cells were dissociated, pooled and re-plated into 5 875 cm^2 multilayer flasks (Falcon 353144). Uninfected iPS cells were dissociated and re-plated at a ratio of 1:3 into a T225 flask. 48 hours after passaging, cells were treated with 0.4 µg/mL puromycin. Puromycin selection media was exchanged daily until no cells remained in the uninfected control condition for a total of 6 days. When the Brunello lentiCRISPR infected iPS cells had reached 80% confluence they were passaged for differentiation and a portion were reserved for DNA collection. Cells were re-plated at a density of 14.5 million cells/ T225 flask and differentiation was performed as described previously.[1] Cells reserved for sequencing were pelleted, rinsed with cold PBS and stored at -20°C. After day 25 of differentiation 116 million cardiomyocytes were re-plated for doxorubicin treatment and the remaining 180 million cells were reserved for DNA collection as the no doxorubicin control. 24 hours after re-plating, unattached cells were removed by rinsing flasks with PBS, and cardiomyocytes were treated with 3 µM doxorubicin. After 72 hours of doxorubicin treatment, cells were dissociated and reserved for DNA collection.

DNA preparation and sequencing:

DNA was extracted from frozen cell pellets using Blood & Cell Culture DNA maxi kit (QIAGEN 13362) for iPS cell and cardiomyocyte control samples and using Blood & Cell Culture DNA midi kit (QIAGEN 13343) for the doxorubicin treated condition. One round of PCR was performed using Ex Taq DNA polymerase (Clontech RR001A) using a pooled, staggered P5 primer and a unique barcoded P7 primer. PCR amplification was performed using 240 µg of DNA for iPS cell and cardiomyocyte control samples, maintaining adequate library representation. These reactions were split into 24 reactions per sample, with 12 reactions for each of two unique P7 barcoded primers. For doxorubicin samples, since there were fewer cells per condition, 6 reactions were performed for each sample using a total of 60 µg of DNA and PCR was performed with 2 unique P7 barcoded primers per sample, with 3 reactions per primer. Amplicons from the PCR reactions were pooled, giving 2 pools per sample, each with a unique P7 barcode. Pooled PCR products were purified using Agencourt AMPure XP (Beckman Coulter A63880) magnetic beads as described here.[3] Purified PCR products were sequenced on an Illumina Hiseq2500 in for 55 cycles in rapid run mode by Jeremy Day at the LIAI Sequencing Core Facility.

CRISPR Screen Analysis:

FASTQ read files were trimmed using the cutadapt algorithm to remove the staggered adapter sequence and sgRNA read counts were obtained using the using the Mageck software package count algorithm [5,6]. Read counts were filtered to include only sgRNAs with at least 50 counts in the no-doxorubicin cardiomyocyte control sample and counts were normalized to the total number of reads and imputed for missing values.

reads per sgRNA/ total number of mapped reads in sample X $10^6 + 1$

The RIGER algorithm extension for GENE-E was employed to analyze sgRNA distribution and rank genes according to enrichment in the doxorubicin condition compared to the no-doxorubicin cardiomyocyte control. [7] Genes represented by only

one sgRNA were excluded from analysis and p-values were calculated using the Kolmogorov-Smirnov method. Gene level representation was calculated from the mean of normalized sgRNA counts.

DAVID gene ontology algorithm was used to perform functional annotation clustering and identify enriched gene-sets for genes lost during iPS cell maintenance and cardiomyocyte differentiation and for the top 100 genes enriched after doxorubicin treatment.[8]

Doxorubicin Toxicity Assay

Cells were incubated with a range of concentrations of doxorubicin (Sigma D1515) in 96 well optical bottom plates (Thermo Fisher 165305) for 72 hours after which cells were fixed in 4% paraformaldehyde (Affymetrics 19943), permeabilized in 0.2% Triton-X in PBS and stained with DAPI (Fisher Scientific 62247). Plates were sealed using adhesive foil (Spectrum SP0028) and imaged on an ImageXpress Micro high-content imaging system and data was processed using the MetaExpress system using the multiwavelength cell scoring algorithm at the Functional Genomics Core at La Jolla Institute for Allergy and Immunology.

Flow cytometry for cardiac marker expression:

iPS derived cardiomyocytes were detached with Accutase and rinsed with PBS. Cells were then fixed in 1% paraformaldehyde (PFA). PBS + 1% BSA (SeraCareAP4510-80) was used for all subsequent wash steps. Cells were stored in 90% methanol until ready to stain. For staining cells were blocked with 10% normal donkey serum (Abcam ab7475) with 0.5% BSA and 0.5% Triton X-100 (Sigma X100). For primary antibody staining cells were incubated with 2µg/mL cTnT primary antibody (Thermo Fisher MA5-12960), 0.1 ug/mL anti-MLC(Abcam ab89594) or in 1% normal donkey serum with 0.5% BSA and 0.05% Triton X-100 in PBS for 45 minutes at 4^oC. For secondary antibody staining incubated with Alexa594 anti mouse secondary antibody for 45 minutes at 4^oC. Flow cytometry analysis was performed on an LSR Fortessa System (BD Biosciences) at the LIAI flow cytometry core.

SLCO1A2 knockdown lines:

Plasmid encoding scramble (VB180213-1353fph) or SLCO1A2 (VB180213-1350ygd; VB180221-1001tjf) targeting shRNAs were purchased from VectorBuilder. Lentivirus was generated as described above. iPS and ES cells were dissociated with Accutase and re-plated at a density of 200,000 cells per well and BT549 breast cancer cells were dissociated with trypsin and re-plated at a density of 500,00 cells per well in a 6-well plate in media containing 8 µg/mL polybrene and 15 µL of shRNA encoding virus. iPS cells expressing the lentiviral cassette were selected using puromycin at a concentration of 0.4 µg/mL for iPS cells and 1 µg/mL for BT549 cells until no cells remained in an untreated control well. iPS cells were expanded and differentiated to cardiomyocytes as described above. The doxorubicin toxicity assay was performed as described above on day 30 post differentiation of iPS and ES derived cardiomyocytes. Pairwise Student’s T-test was performed to assess statistical significance between scramble shRNA and SLCO1A2 targeting shRNAs.

SLCO1A2 overexpressing lines:

Plasmid encoding for SLCO1A2 over-expression (Genscript OHu23414D) under the CMV promoter was transfected into HEK cells. HEK293T cells were plated in 6 well plates 40% confluence 24 hours before plasmid transfection. Transfection was performed in OptiMem medium supplemented with Lipofectamine and Plus Reagent. Cells were mock transfected without plasmid for the no plasmid control condition. Statistical significance was assessed using pairwise ttest between SLCO1A2 over-expressing plasmid and no plasmid control. Cells transiently expressing SLCO1A2 were subjected to the doxorubicin toxicity assay described above.

Plasmid encoding for stable SLCO1A2 over-expression (VB180531-1392anf) under control of the CMV promoter was purchased from VectorBuilder and lentivirus was generated as described above. HEK cells were dissociated with Trypsin and re-plated at a density of 500,000 cells per well in a 6-well plate in media containing 8ug/mL polybrene and 20 µL of SLCO1A2 encoding virus. HEK cells expressing SLCO1A2 were selected using 1 µg/mL puromycin.

Doxorubicin uptake assay:

HEK cells stably overexpressing SLCO1A2 or control HEK cells were treated with 3 µM doxorubicin in Hank's Balanced Salt Solution (Thermo Fisher 14025076) for 15 minutes at 37°C. Doxorubicin treated cells were washed once with PBS and cells were collected in 450 µL of ethanol (Sigma 459828) followed by addition of 112.5 µL LCMS grade water (Fisher Scientific W6-4). Cells were subsequently lysed by three freeze-thaw cycles by alternating samples between a 37°C water bath and a -80°C dry ice and acetone (Praxair UN1845) bath in 30 second intervals. Insoluble protein was allowed to precipitate at -20°C for 30 minutes and pelleted by centrifugation at 14000 RPM at 4°C for 10 minutes. Supernatant was removed and solvent was evaporated using a vacuum concentrator (Thermo Fisher RVT5105) and resulting sample was resuspended in 75% water, 20% ethanol, 5% acetonitrile (Sigma 34998).

Cell lysate was analyzed as reported previously using a Thermo Vanquish ultra-pressure liquid-chromatography (UPLC) system paired with a Thermo QExactive HF Hybrid Quadrupole-Orbitrap Mass Spectrometer (Thermo Fisher IQLAAEGAAPFALGMBFZ)[9]. Separation was performed using a Phenomenex Kinetex C18 column (100x2.1mm, 1.7mm, P/N 00D-4475-AN). Data was processed using MZmine 2 (http://mzmine.github.io/) to extract the LCMS peak height for doxorubicin to determine the relative amount of doxorubicin present in the cell lysates [10]. Doxorubicin content was normalized to that of control HEK cells.

Western-blotting:

Heart tissue protein lysate was obtained from explanted human cardiac tissue. Protein was extracted from iPS derived cardiomyocytes after 25 days of differentiation using MPER (Thermo Fisher 78501). Western blotting was performed under reducing conditions using NuPage 4-12% Bis-Tris Gels (Thermo Fisher NP0327BOX) on a Bolt Mini Gel Tank (Thermo Fisher A25377) using MOPS Buffer (Thermo Fisher NP000102).

Transfer was performed using a Mini Blot Module (Thermo Fisher B1000 to transfer protein onto a 0.2 µm PVDF membrane (Thermo Fisher LC2002) NuPage transfer buffer (NP00061). Blots were blocked in 5% dry milk, were incubated with primary antibody against SLCO1A2 or SLCO1B3 and subsequently incubated with HRP conjugated secondary antibody. Detection was performed with Pierce ECL Western Blotting Substrate (Thermo Fisher 32106) and ECL Hyperfilms (GE 28906838).

Karyotype analysis:

G-band karyotype analysis with a band resolution of 350-375 was performed on untransduced, baseline iPS cells at passage 20 by WiCell Research Institute.

RT qPCR:

Analysis of gene expression levels was performed using reverse transcription (Qiagen 205413) followed by real-time, quantitative PCR using SYBR Green (204145) on a CFX Touch RT PCR system (BioRad 1855196).

Primers:

Primer Name	Primer Sequence
P5_0nt_stagger	5'AATGATACGGCGACCACCGAGATCTACACTCTTTCCCTACACGACGCTCTTCCGATCTTTGTGGAAAGGACGAAACACCG
P5_1nt_stagger	5'AATGATACGGCGACCACCGAGATCTACACTCTTTCCCTACACGACGCTCTTCCGATCTCTTGTGGAAAGGACGAAACACCG
P5_2nt_stagger	5'AATGATACGGCGACCACCGAGATCTACACTCTTTCCCTACACGACGCTCTTCCGATCTGCTTGTGGAAAGGACGAAACACCG
P5_3nt_stagger	5'AATGATACGGCGACCACCGAGATCTACACTCTTTCCCTACACGACGCTCTTCCGATCTAGCTTGTGGAAAGGACGAAACACCG
P5_4nt_stagger	5'AATGATACGGCGACCACCGAGATCTACACTCTTTCCCTACACGACGCTCTTCCGATCTCAACTTGTGGAAAGGACGAAACACCG
P5_6nt_stagger	5'AATGATACGGCGACCACCGAGATCTACACTCTTTCCCTACACGACGCTCTTCCGATCTTGCACCTTGTGGAAAGGACGAAACACCG
P5_7nt_stagger	5'AATGATACGGCGACCACCGAGATCTACACTCTTTCCCTACACGACGCTCTTCCGATCTACGCAACTTGTGGAAAGGACGAAACACCG
P5_8nt_stagger	5'AATGATACGGCGACCACCGAGATCTACACTCTTTCCCTACACGACGCTCTTCCGATCTGAAGACCCTTGTGGAAAGGACGAAACACCG
P7 primer lentiCRISPRv2-1	5'CAAGCAGAAGACGGCATACGAGATCGGTTCAAGTGACTGGAGTTCAGACGTGTGCTCTTCCGATCTCCAATTCCCACTCCTTTCAAGACCT
P7 primer lentiCRISPRv2-2	5'CAAGCAGAAGACGGCATACGAGATGCTGGATTGTGACTGGAGTTCAGACGTGTGCTCTTCCGATCTCCAATTCCCACTCCTTTCAAGACCT

P7 primer lentiCRISPRv2-3	5’CAAGCAGAAGACGGCATACGAGATTAACTCGGGTGACTGGAGTTCAGACGTGTGCTCTTCCGATCTCCAATTCCCACTCCTTTCAAGACCT
P7 primer lentiCRISPRv2-4	5’CAAGCAGAAGACGGCATACGAGATTAACAGTTGTGACTGGAGTTCAGACGTGTGCTCTTCCGATCTCCAATTCCCACTCCTTTCAAGACCT
P7 primer lentiCRISPRv2-5	5’CAAGCAGAAGACGGCATACGAGATATACTCAAGTGACTGGAGTTCAGACGTGTGCTCTTCCGATCTCCAATTCCCACTCCTTTCAAGACCT
P7 primer lentiCRISPRv2-6	5’CAAGCAGAAGACGGCATACGAGATGCTGAGAAGTGACTGGAGTTCAGACGTGTGCTCTTCCGATCTCCAATTCCCACTCCTTTCAAGACCT
P7 primer lentiCRISPRv2-9	5’CAAGCAGAAGACGGCATACGAGATCGGTGACCGTGACTGGAGTTCAGACGTGTGCTCTTCCGATCTCCAATTCCCACTCCTTTCAAGACCT
P7 primer lentiCRISPRv2-10	5’CAAGCAGAAGACGGCATACGAGATTACAGAGGGTGACTGGAGTTCAGACGTGTGCTCTTCCGATCTCCAATTCCCACTCCTTTCAAGACCT
ACTB F1	5’CCTTGCACATGCCGGAG3’
ACTB R1	5’ACAGAGCCTCGCCTTTG3’
SLCO1A2 F1	5’TTTCAGGCAACTTGTCCTCA3’
SLCO1A2 R1	5’GTACACCCACATTAATGATTTAACTTC3’
POU5F1 F1	5’GTTGGAGGGAAGGTGAAGTTC3’
POU5F1 R1	5’TGTGTCTATCTACTGTGTCCCA3’
NANOG F1	5’CCTTCTGCGTCACACCATT3’
NANOG R1	5’AACTCTCCAACATCCTGAACC3’
SOX2 F1	5’GTACAACTCCATGACCAGCTC3’
SOX2 R1	5’CTTGACCACCGAACCCAT3’

Supplementary References:

1 Lian, X. *et al.* Robust cardiomyocyte differentiation from human pluripotent stem cells via temporal modulation of canonical Wnt signaling. *Proc Natl Acad Sci U S A* **109**, E1848-1857, doi:10.1073/pnas.1200250109 (2012).

2 Tohyama, S. *et al.* Distinct metabolic flow enables large-scale purification of mouse and human pluripotent stem cell-derived cardiomyocytes. *Cell Stem Cell* **12**, 127-137, doi:10.1016/j.stem.2012.09.013 (2013).

3 Doench, J. G. *et al.* Optimized sgRNA design to maximize activity and minimize off-target effects of CRISPR-Cas9. *Nat Biotechnol* **34**, 184-191, doi:10.1038/nbt.3437 (2016).

4 Shalem, O. *et al.* Genome-scale CRISPR-Cas9 knockout screening in human cells. *Science* **343**, 84-87, doi:10.1126/science.1247005 (2014).

5 Martin, M. Cutadapt removes adapter sequences from high-throughput sequencing reads. *EMBnet.journal* **17**, 10-12, doi:10.14806/ej.17.1.200 (2011).
6 Li, W. *et al.* MAGeCK enables robust identification of essential genes from genome-scale CRISPR/Cas9 knockout screens. *Genome Biol* **15**, 554, doi:10.1186/s13059-014-0554-4 (2014).
7 Luo, B. *et al.* Highly parallel identification of essential genes in cancer cells. *Proc Natl Acad Sci U S A* **105**, 20380-20385, doi:10.1073/pnas.0810485105 (2008).
8 Huang da, W., Sherman, B. T. & Lempicki, R. A. Systematic and integrative analysis of large gene lists using DAVID bioinformatics resources. *Nat Protoc* **4**, 44-57, doi:10.1038/nprot.2008.211 (2009).
9 Lagerborg, K. A., Watrous, J. D., Cheng, S. & Jain, M. High-Throughput Measure of Bioactive Lipids Using Non-targeted Mass Spectrometry. *Methods Mol Biol* **1862**, 17-35, doi:10.1007/978-1-4939-8769-6_2 (2019).
10 Pluskal, T., Castillo, S., Villar-Briones, A. & Oresic, M. MZmine 2: modular framework for processing, visualizing, and analyzing mass spectrometry-based molecular profile data. *BMC Bioinformatics* **11**, 395, doi:10.1186/1471-2105-11-395 (2010).

Supplementary Figure 1

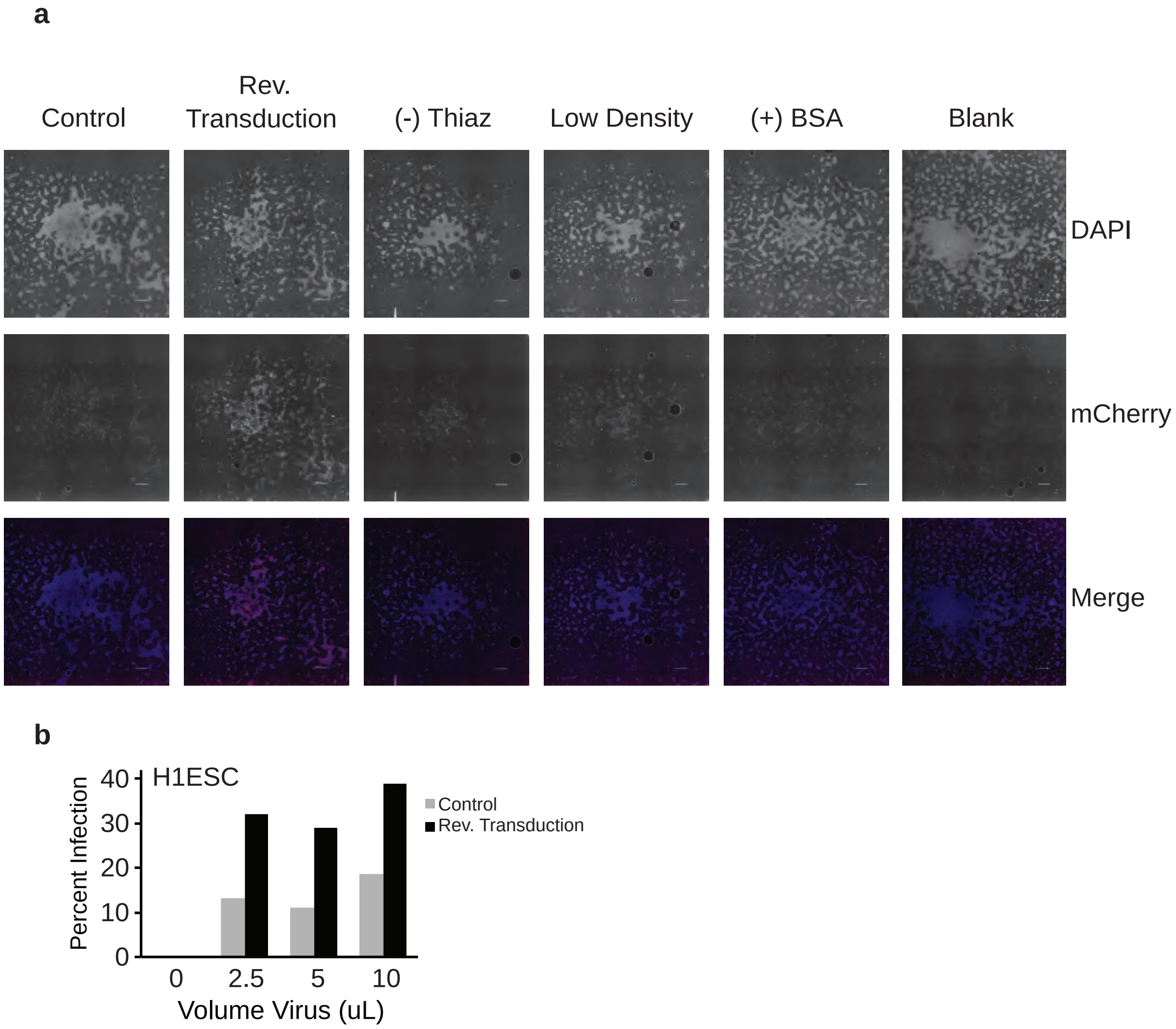

Supplementary Figure 1: Optimizing CRISPR/Cas9 transfer in human stem cells.
(a) Optimization of infection efficiency with mCherry expressing virus. Scale bar = 400 μm
(b) Infection Efficiency in H1ESC line using standard (control) or reverse transduction approaches (n=2 per condition).

Supplementary Figure 2

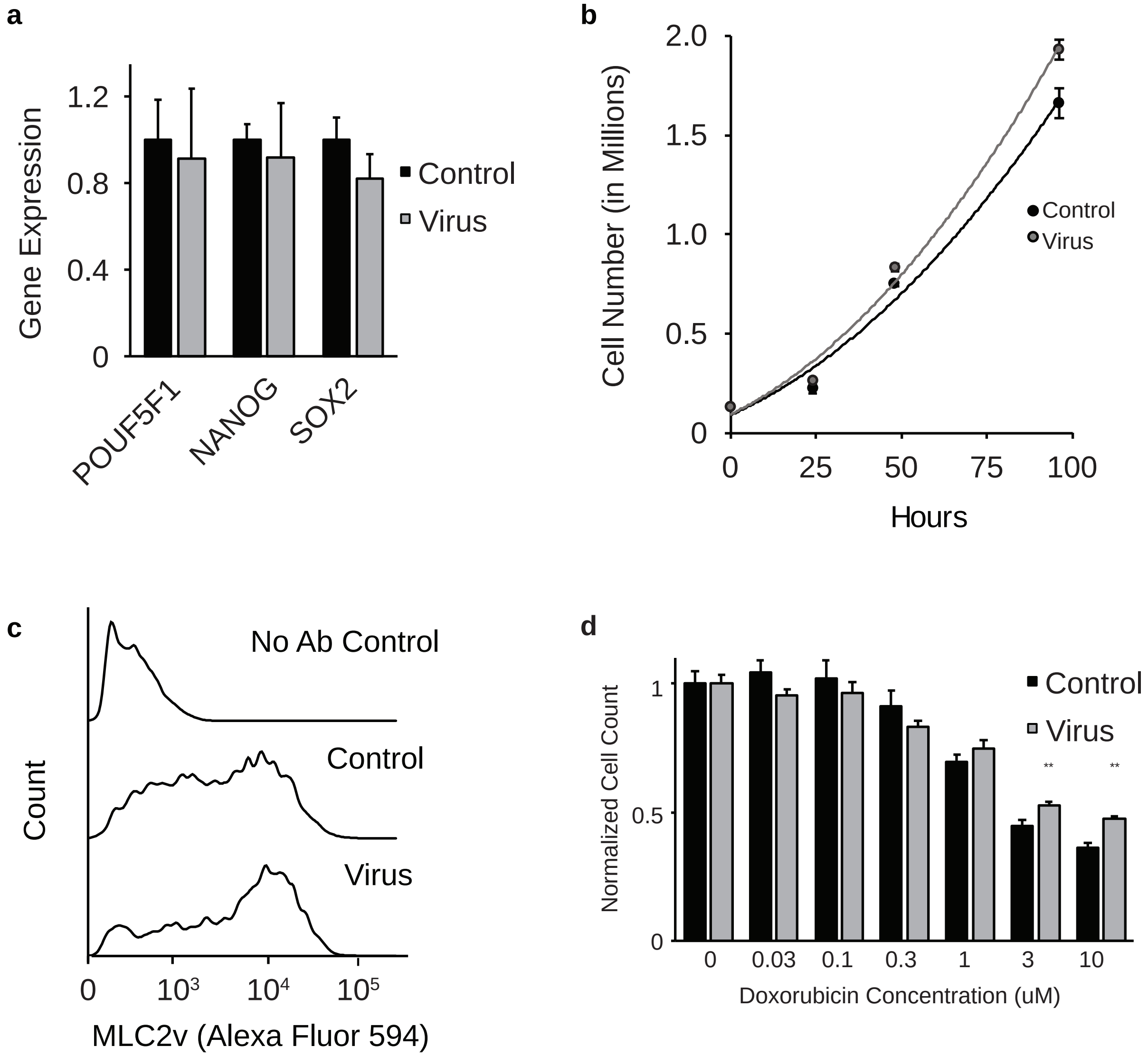

Supplementary Figure 2: Phenotypic resilience in pooled CRISPR-Cas9 infected stem cells. Uninfected H1ESCs (control) or those transduced with CRISPR/Cas9 library (virus) were assessed for **(a)** expression of pluripotency markers (n=3 per condition) and **(b)** proliferative potential (n=3 per condition). Uninfected iPS cells (control) or those transduced with CRISPR/Cas9 library (virus) were assessed for **(c)** myosin light chain 2 (MLC2) protein expression and **(d)** doxorubicin dose response after differentiation to cardiomyocytes . (**: $p < 0.01$). p from Student's T-Test.

Supplementary Figure 3

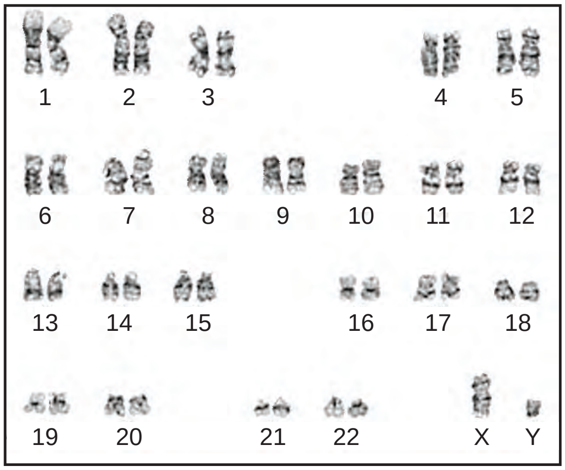

Supplementary Figure 3: Karyotype analysis of untreated iPS Cells. Uninfected iPS cells were analyzed for abnormalities in chromosome number or chromosonal structure using karyotype analysis.

Supplementary Figure 4

a

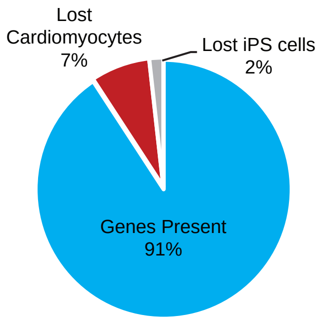

b

Enrichment Analysis Annotation Clusters			
iPS cells		Cardiomyocytes	
Ribosomal Proteins	4.58	RNA Splicing	4.51
RNA Elongation	2.28	mRNA Processing	3.47
tRNA Biosynthesis	1.95	Translation	2.77

Supplementary Figure 4: Gene-level Library Representation in iPS cells and Cardiomyocytes. (a) Percentage of genes present with 2 or more sgRNAs at more than 50 read-counts (Genes Present) or less than 2 sgRNAs (Lost) at either the iPS and Cardiomyocyte stage of the screen. **(b)** DAVID gene ontology enrichment analysis for those genes with less than 2

Supplementary Figure 5

Enrichment Analysis Annotation Clusters	
Cluster Annotation	Enrichment Score
Iron Metabolism	1.44
Membrane Proteins	0.85
Mitochondrial Proteins	0.60

Supplementary Figure 5: Analysis for Genes Enriched in Doxorubicin Treatment. DAVID gene ontology clustering analysis results for the top 100 enriched genes by fold enrichment in doxorubicin treated versus untreated control cardiomyocytes.

Supplementary Figure 6

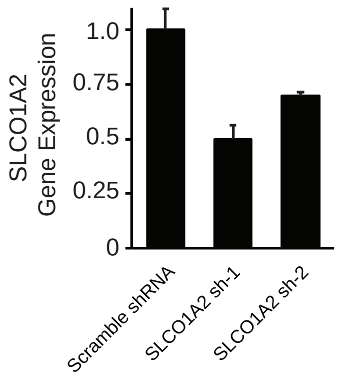

Supplementary Figure 6: SLCO1A2 Knockdown in shRNA treated iPS cells. SLCO1A2 gene expression in iPSCs treated with scramble or SLCO1A2 targeting shRNAs (n=3 per condition).

Supplementary Figure 7

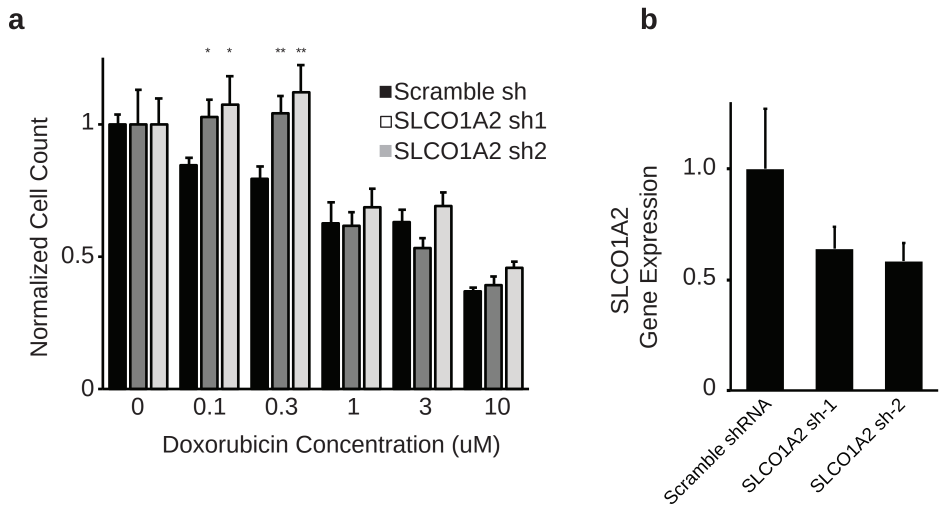

Supplementary Figure 7: Doxorubicin Toxicity in SLCO1A2 Knockdown Human ES Cell Derived Cardiomyocytes. (a) Doxorubicin dose response in SLCO1A2 shRNA knockdown H1ESC derived cardiomyocytes (n=8 per condition) (*: $p < 0.05$, **: $p < 0.01$). p from Student's T-Test. **(b)** SLCO1A2 gene expression in H1ESCs treated with scramble or SLCO1A2 targeting shRNAs (n=3 per condition).

Supplementary Figure 8

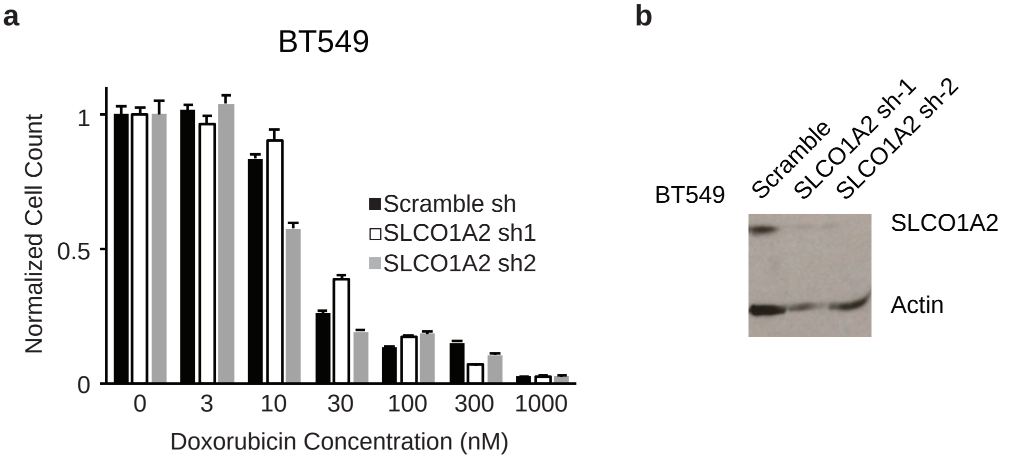

Supplementary Figure 8: Doxorubicin Toxicity in SLCO1A2 Knockdown Breast Cancer Cells. (a) Doxorubicin dose response in SLCO1A2 shRNA knockdown BT549 breast cancer cells (n=8 per condition). **(b)** Western Blot of protein level expression of SLCO1A2 in BT549 cells treated with scramble or SLCO1A2 targeting shRNAs. Full length blots are included in Supplementary Figure 10e.

Supplementary Figure 9

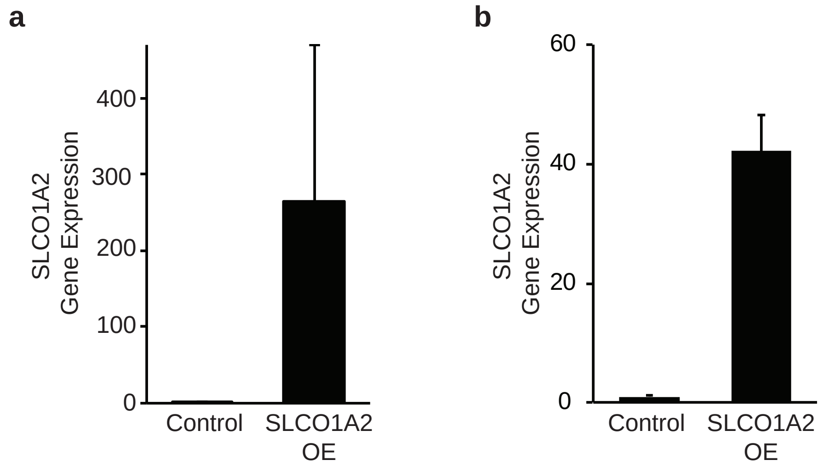

Supplementary Figure 9: SLCO1A2 Over-expression in HEK293T cells. (a) SLCO1A2 gene expression in HEK cells transiently over-expressing SLCO1A2 or mock transfected control (n=3 per condition). **(b)** SLCO1A2 gene expression in cells stably over-expressing SLCO1A2 (n=3 per condition).

Supplementary Figure 10

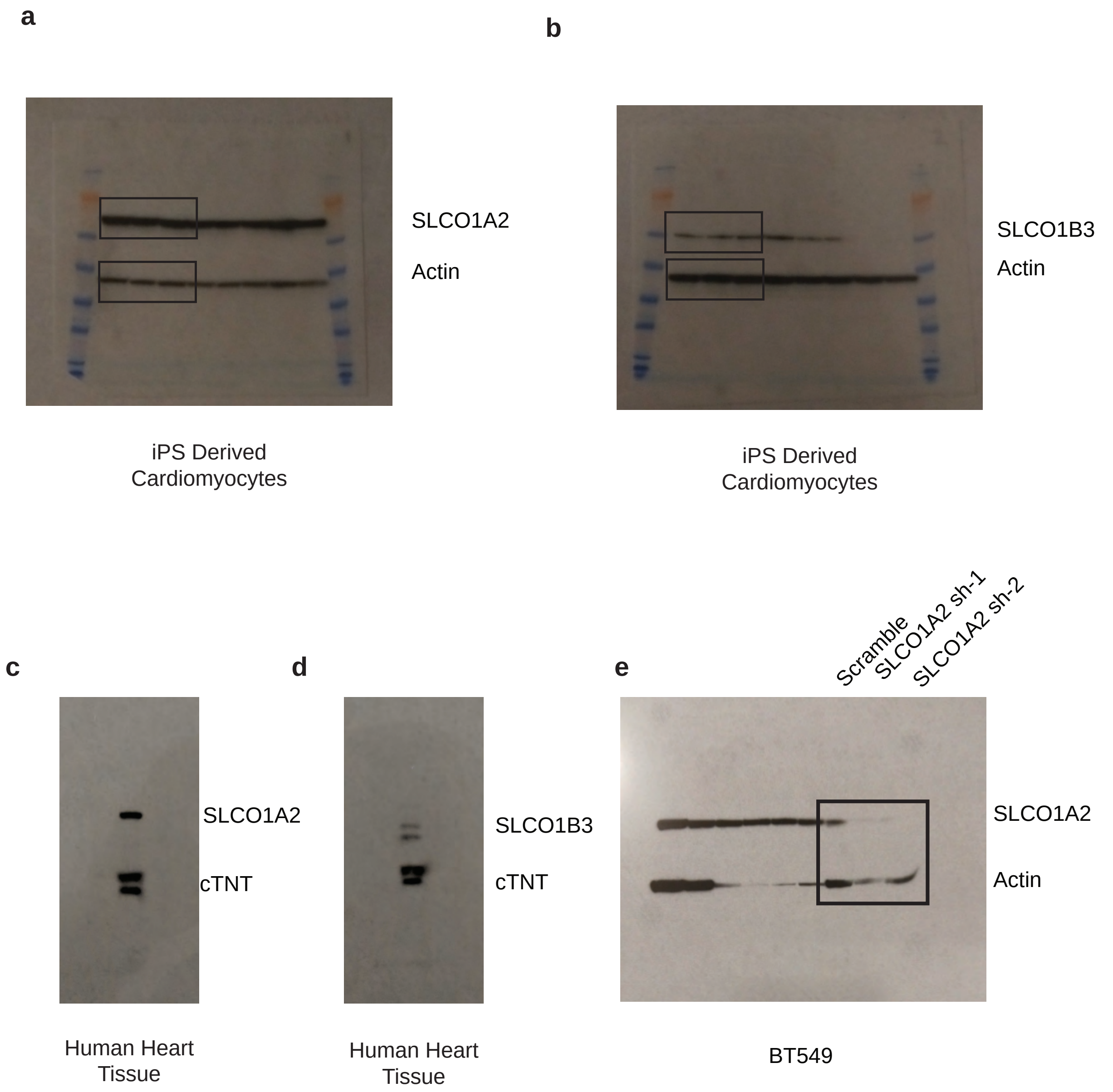

Supplementary Figure 10: SLCO1A2 and SLCO1B3 Expression in iPS derived cardiomyocytes and Breast Cancer Cells. (a) Full length western blot for SLCO1A2 expression in iPS derived cardiomyocytes for corresponding Figure 3e. **(b)** Full length western blot for SLCO1B3 expression in iPS derived cardiomyocytes for corresponding Figure 3e. **(c)** Full length western blot of SLCO1A2 in human heart tissue or corresponding Figure 3e. Blot was trimmed prior to hybridization. **(d)** Full length western blot for SLCO1B3 in human heart tissue or corresponding Figure 3e. Blot was trimmed prior to hybridization. **(e)** Full length western blot of protein level expression of SLCO1A2 in BT549 cells treated with scramble or SLCO1A2 targeting shRNAs for corresponding Supplementary Figure 7b.
